# Minimally invasive lateral lumbar interbody fusion with direct psoas visualization

**DOI:** 10.1186/1749-799X-9-20

**Published:** 2014-03-26

**Authors:** Philip S Yuan, Kasra Rowshan, Rohit B Verma, Larry E Miller, Jon E Block

**Affiliations:** 1 Memorial Orthopedic Surgical Group, 2760 Atlantic Ave, Long Beach, CA 90806, USA; 2 University Orthopaedic Associates, 611 Northern Blvd, Suite 200, Great Neck, NY 11021, USA; 3 Miller Scientific Consulting, Inc, 1854 Hendersonville Road, #231, Asheville, NC 28803, USA; 4 The Jon Block Group, 2210 Jackson Street, Suite 401, San Francisco, CA 94115, USA

**Keywords:** Fusion, Lateral, Lumbar, Minimally invasive, Psoas

## Abstract

**Background:**

Minimally invasive lateral approaches to the lumbar spine have been adopted to allow access to the intervertebral disc space while avoiding the complications associated with anterior or posterior approaches. This report describes a minimally invasive technique for lateral lumbar interbody fusion LLIF that allows direct intraoperative visualization of the psoas and surrounding neurovasculature (DV-LIF).

**Methods:**

The technique utilizes a radiolucent tubular retractor and a secondary psoas retractor that allows a muscle-sparing approach while offering excellent visualization of the operative site. The unique advantage of this procedure is that the psoas muscle and surrounding nerves can be directly visualized intraoperatively to supplement neuromonitoring. We retrospectively reviewed complication rates in 34 patients treated with DV-LLIF (*n* = 19) or standard lateral lumbar interbody fusion (S-LLIF, *n* = 15).

**Results:**

There were 29 complications (median: 1 per patient) with DV-LLIF and 20 (median: 1 per patient) complications with S-LLIF. Postoperative sensory deficits were reported in eight (42%) and seven (47%) patients, respectively. Thigh pain or numbness was reported in eight (42%) and five (33%) patients, respectively. The percentage of the overall complications directly attributable to the procedure was 69% with DV-LLIF and 83% with S-LLIF. One severe complication (back pain) was reported in one DV-LLIF patient and four severe complications (severe bleeding, respiratory failure, deep venous thrombosis and gastrointestinal prophylaxis, and nicked renal vein and aborted procedure) were reported in two S-LLIF patients.

**Conclusions:**

Preliminary evidence suggests that minimally invasive lateral interbody fusion with direct psoas visualization may reduce the risk for severe procedural complications.

## Background

Fusion surgery is a viable treatment option for reducing pain and improving function in patients with chronic low back pain refractory to nonsurgical care. Several open and minimally invasive lumbar fusion approaches are available to the spine surgeon including anterior lumbar interbody fusion (ALIF), posterior lumbar interbody fusion (PLIF), and transforaminal lumbar interbody fusion (TLIF). Iatrogenic injury is an inherent risk of these procedures. ALIF endangers major organs and blood vessels [1-3], while PLIF [4-7] and TLIF [8,9] can cause musculoligamentous injury, nerve root injury, and spinal fluid leakage.

Minimally invasive lateral approaches to the lumbar spine have been adopted to allow access to the intervertebral disc space while avoiding the complications associated with anterior or posterior approaches. The main limitation of lateral approaches is the potential for transient motor and sensory disturbances due to the inability to directly visualize the psoas muscle and the nerves of the lumbosacral plexus [10,11]. The wide variability in lumbar plexus anatomy complicates identification of a safe working zone under fluoroscopy [12], and access to L4–5 is further complicated by longer nerve roots [13] and significant narrowing of the working zone [14-16], resulting in higher complication rates [17]. The incidence of postoperative thigh pain or weakness with lateral interbody fusion using continuous neuromonitoring ranges from 67%–75% [10,18] with some cases lingering for 1 year or more [10]. Even in patients with no significant changes in electromyographic response, motor deficits following transpsoas fusion have been reported in 24% of patients [10]. Lateral approaches for lumbar interbody fusion that allow direct visualization of the psoas and surrounding nerves may improve patient safety.

## Methods

This report describes a minimally invasive technique for lateral interbody fusion (DV-LLIF) that allows direct intraoperative visualization of the psoas and surrounding neurovasculature (VEO Lateral System, Baxano Surgical, Raleigh, NC, USA). This technique utilizes a radiolucent tubular retractor and an internal psoas retractor that allows a muscle-sparing approach while offering excellent visualization of the operative site [19].

**Figure 1:**
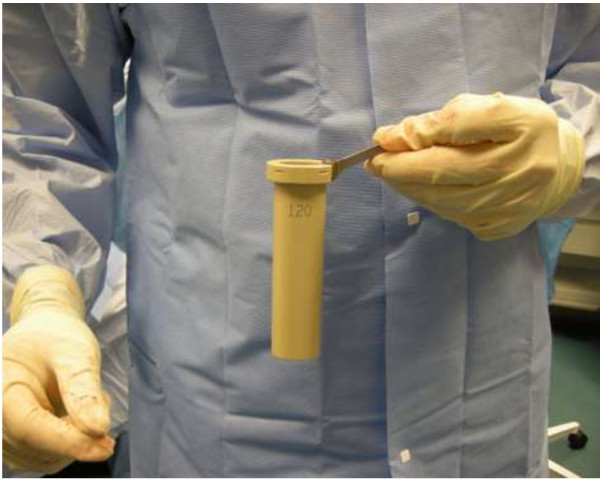
Fixed radiolucent tube allows for direct psoas visualization and improves fluoroscopic visibility.

**Figure 2:**
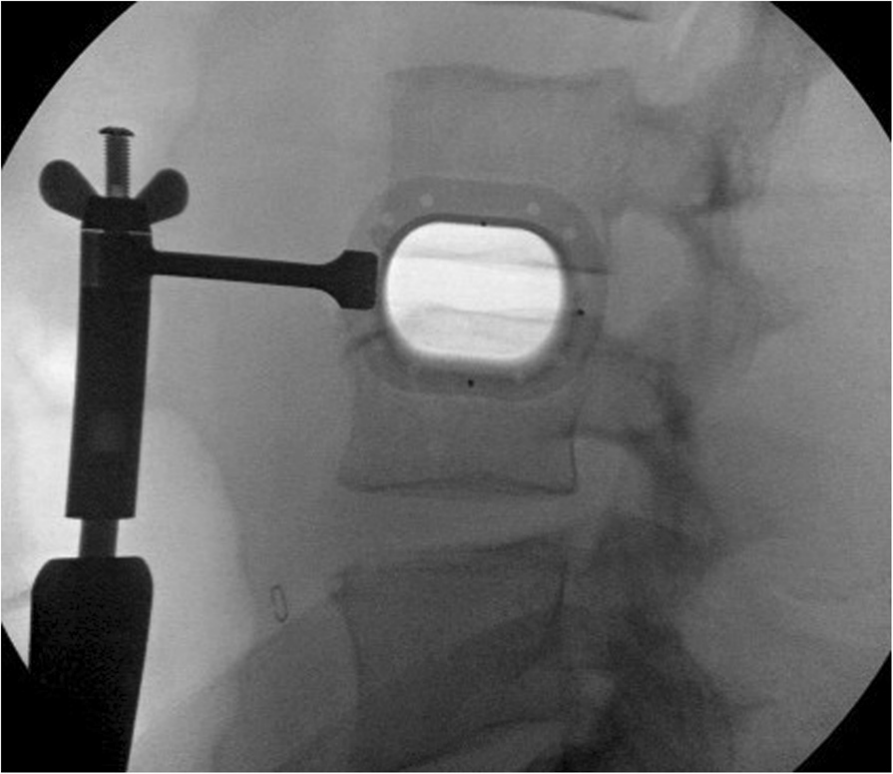
Fluoroscopic view demonstrating optimal radiolucent tube positioning directly over disc space.

**Figure 3:**
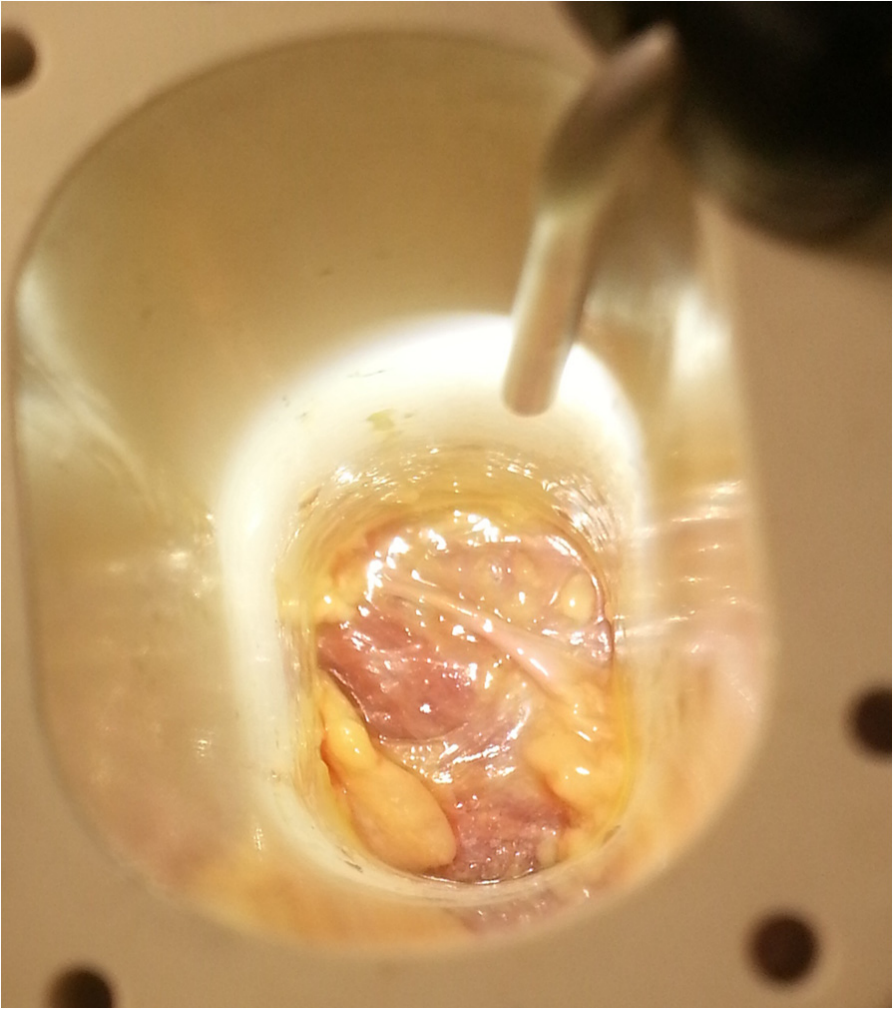
Direct psoas and nerve visualization.

**Figure 4:**
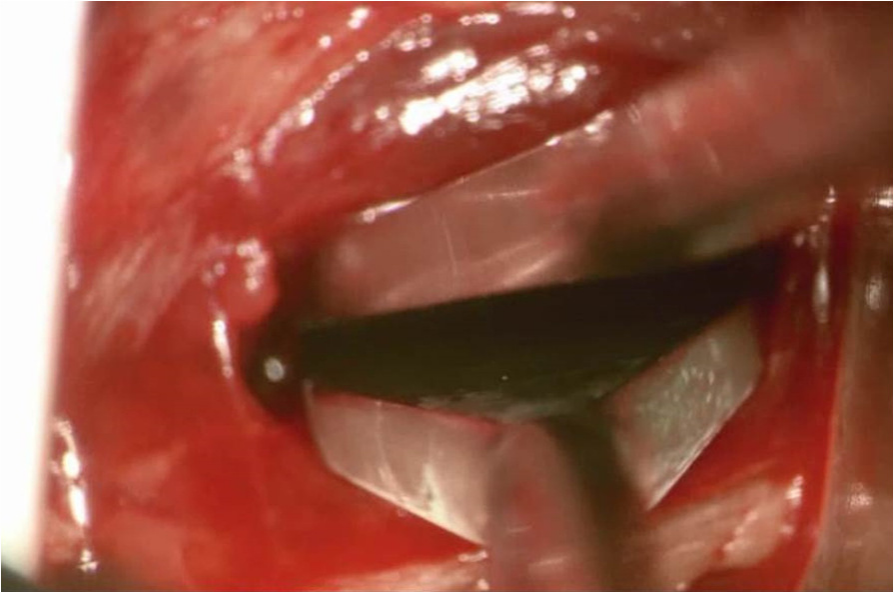
Manual psoas dissection.

**Figure 5:**
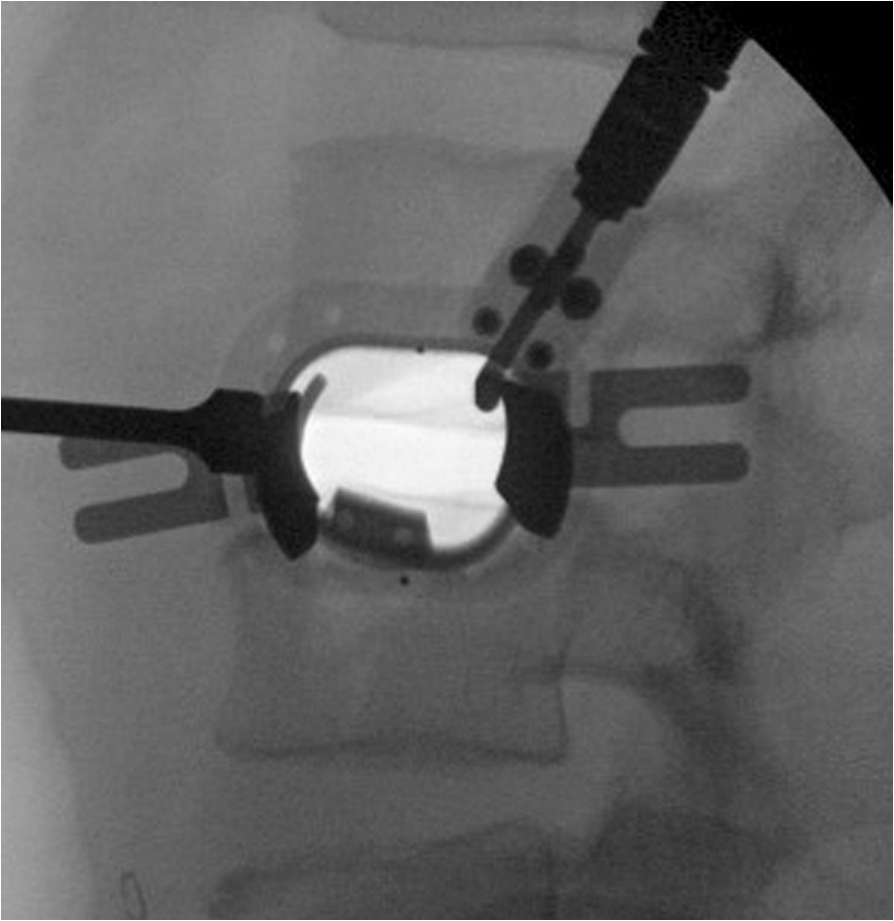
Lateral fluoroscopic image of fully-assembled DV-LIF retractor.

With the patient in the lateral decubitus position, true anteroposterior and lateral fluoroscopic images are obtained. A 3-cm anteroposterior incision is made over the center of the disc space, and dissection is continued deep to the external oblique fascia. The muscle layers of the abdominal wall are longitudinally separated with blunt instruments to access the retroperitoneal space. The surgical corridor is then established using sequential blunt dilators inserted through the retroperitoneal space and onto the surface of the psoas muscle. Once correct retractor position is confirmed with fluoroscopy, a radiolucent tube (Figure 1) is inserted, the dilators are removed, and a lateral fluoroscopic image confirms correct positioning over the disc space (Figure 2). At this point in the procedure, the psoas muscle and surrounding nerves can be directly visualized (Figure 3). Dissection continues in the anteroposterior direction to the level of the disc (Figure 4). The nerve roots may be directly visible through the tube prior to and during dissection. Depending of the level treated, the sensory root may be observed traversing the surface of the muscle, while the motor roots are most commonly observed to the posterior. Two hand-held blades and an inner sleeve are used to retract the psoas muscle to the level of the disc an annulotomy is performed (Figure 5). Discectomy and endplate preparation are then completed using standard instruments. Autogenous bone graft material is packed inside a PEEK implant, which is inserted into the disc space. Final anteroposterior and lateral images are taken, all instrumentation is removed, and the wound is closed in the standard fashion. The technique is indicated for use with supplemental fixation devices, which may be selected at the surgeon's discretion.

## Results and discussion

The evidence that direct visualization of the psoas during LLIF reduces iatrogenic complications is limited, yet promising. We retrospectively reviewed 34 cases (Table 1) treated with DV-LLIF (*n* = 19) or (S-LLIF, *n* = 15) for degenerative disc disease. Following IRB approval at each center, retrospective chart reviews were performed at four centers with experience in both techniques for consecutive patients treated with DV-LLIF or S-LLIF between October 2011 and August 2013.

**Table 1:** Baseline characteristics of 34 cases treated with DV-LLIF or S-LLIF

**Variable**	**DV-LLIF** ***n*** **= 19**	**S-LLIF** ***n*** **= 15**	***P*** **value**
Male gender, *n* (%)	6 (32)	11 (73)	0.04^a^
Age, year, median (IQR)	65 (53–70)	66 (53–74)	0.87^b^
Body mass index, kg/m^2^, median (IQR)	29 (23–34)	27 (22–29)	0.27^b^

^a^Fisher's exact test; ^b^Mann-Whitney *U* test. *IQR* interquartile range.

All complications, regardless of severity, were recorded into a pre-defined database (Table 2). Complications were categorized as mild, moderate, or severe. A mild complication was transient or caused mild discomfort, required no intervention/therapy, and did not interfere with the normal activities. A moderate complication caused some limitation in activity, which may have required assistance, and no or minimal intervention/therapy was required. A severe complication caused marked limitation in activity, interrupted usual daily activity, and required medical intervention/therapy.

**Table 2:** Complications in 34 cases treated with DV-LLIF or S-LLIF

**Complication data**	**DV-LLIF** ***n*** **= 19**	**S-LLIF** ***n*** **= 15**	***P*** **value**
Patients with any complication, *n* (%)	15 (79)	12 (80)	0.64^a^
Complications per patient, median (IQR)	1.0 (0.5–1.5)	1.0 (1.0–2.0)	0.25^b^
Severity, *n*			
Mild	19	11	
Moderate	9	5	
Severe	1	4	0.10^a,c^

^a^Fisher's exact test; ^b^Mann-Whitney *U* test; ^c^*P* value calculated for severe complications. *IQR* interquartile range.

There were 29 complications (median, 1.0 per patient) with DV-LLIF and 20 (median, 1.0 per patient) complications with S-LLIF. Postoperative sensory deficits were reported in eight (42%) and seven (47%) patients, respectively. Thigh pain or numbness was reported in eight (42%) and five (33%) patients, respectively. The percentage of overall complications directly attributable to the procedure was 69% with DV-LLIF and 83% with S-LLIF. A severe complication (back pain at day 70) was reported in one (5%) patient with DV-LLIF, while four severe complications (severe bleeding, respiratory failure, deep venous thrombosis and gastrointestinal prophylaxis, and nicked renal vein and aborted procedure) were reported in two (13%) patients treated with S-LLIF. Median time to complication was 30 (IQR, 11–61) days with DV-LLIF and 24 (0–75) days with S-LLIF.

The reductions in procedural risks with DV-LLIF have been reported by others. Hardenbrook [20] reported no nerve, vascular, or intra-abdominal injuries and one case of transient lower extremity weakness in 65 subjects (87 levels). Fleischer and colleagues [21] treated 27 patients with LLIF using direct visualization. All cases were technically successful and patients treated had lower complication rates compared to open fusion controls, including overall complications, pain, paresthesias, motor weakness, and need for thigh anesthesia.

Although this research was limited by a small sample size, the thoroughness of complication reporting is a strength of the paper since we reported all complications, regardless of the severity. This is likely why the complication rates are higher than those typically reported for lumbar interbody fusion. Considering that only ‘severe’ complications required treatment, the reported rates of 5% for DV-LLIF and 13% for S-LLIF are comparable to previous literature [17,22].

## Conclusions

Based on preliminary data, a minimally invasive LLIF technique that allows direct visualization of the operative field may reduce the risk for severe procedural complications. Additionally, direct visualization of the operative field allows the surgeon the opportunity to abort the procedure if the surgical corridor involves unanticipated anatomical obstructions. As with any surgical procedure, spine surgeons must be intimately familiar with relevant anatomy, and thorough training and experience with the transpsoas approach are paramount to achieving optimal clinical results.

## Nomenclature

LLIF = lateral lumbar interbody fusion

## Study groups

Direct visualization LLIF (DV-LLIF) vs. standard LLIF (S-LLIF)

## Competing interests

LEM and JEB received financial support from Baxano Surgical, Inc. for assistance with manuscript development.

## Authors' contributions

LM wrote the first manuscript draft. PY, KR, RV, and JB provided critical review and revision. All authors read and approved the final manuscript.
